# Harnessing the power of advertising to prevent childhood obesity

**DOI:** 10.1186/1479-5868-10-114

**Published:** 2013-10-04

**Authors:** Andrew Colin Bell, Luke Wolfenden, Rachel Sutherland, Lucy Coggan, Kylie Young, Michael Fitzgerald, Rebecca Hodder, Neil Orr, Andrew J Milat, John Wiggers

**Affiliations:** 1 School of Medicine and Public Health, University of Newcastle, Newcastle, Australia; 2 Hunter New England Local Health District - Population Health, Locked Bag 10, Wallsend, NSW 2287, Australia; 3 Social Marketing/Communication Consultant, Concord, NSW 2137, Australia; 4 NSW Ministry of Health, Centre for Epidemiology and Evidence, Locked Mail Bag 961, North Sydney, NSW 2059, Australia; 5 School of Public Health, The University of Sydney, Sydney, Australia

## Abstract

**Background:**

Social marketing integrates communication campaigns with behavioural and environmental change strategies. Childhood obesity programs could benefit significantly from social marketing but communication campaigns on this issue tend to be stand-alone.

**Methods:**

A large-scale multi-setting child obesity prevention program was implemented in the Hunter New England (HNE) region of New South Wales (NSW), Australia from 2005–2010. The program included a series of communication campaigns promoting the program and its key messages: drinking water; getting physically active and; eating more vegetables and fruit. Pre-post telephone surveys (n = 9) were undertaken to evaluate awareness of the campaigns among parents of children aged 2–15 years using repeat cross-sections of randomly selected cohorts. A total of 1,367 parents (HNE = 748, NSW = 619) participated.

**Results:**

At each survey post baseline, HNE parents were significantly more likely to have seen, read or heard about the program and its messages in the media than parents in the remainder of the state (p < 0.001). Further, there was a significant increase in awareness of the program and each of its messages over time in HNE compared to no change over time in NSW (p < 0.001). Awareness was significantly higher (p < 0.05) in HNE compared to NSW after each specific campaign (except the vegetable one) and significantly higher awareness levels were sustained for each campaign until the end of the program. At the end of the program participants without a tertiary education were significantly more likely (p = 0.04) to be aware of the brand campaign (31%) than those with (20%) but there were no other statistically significant socio-demographic differences in awareness.

**Conclusions:**

The Good for Kids communication campaigns increased and maintained awareness of childhood obesity prevention messages. Moreover, messages were delivered equitably to diverse socio-demographic groups within the region.

## Background

Television advertising is a powerful medium, able to influence behaviour change by providing information in a relevant and engaging way [1]. It has the ability to achieve mass exposure at a cost that is affordable, [2] and is a major vehicle for the mass marketing of energy dense nutrient poor foods and beverages to consumers. [3-6] In response to the marketing power of commercial industries promoting unhealthy products, governments and public health practitioners have adopted similar broadcast advertising techniques to promote healthy behaviours [7]. Such techniques have helped change social norms to encourage tobacco control and prevention of HIV/AIDS, with the most effective techniques being those linked to broader behavioural and environmental change strategies [8].

Given international concerns about obesity prevalence, [9] communication campaigns centred on television advertising have increasingly been applied to promoting healthy eating and physical activity. However, they have tended to be stand-alone, [10,11] and not true social marketing campaigns, [12] linked to broader strategies providing people with the opportunity to learn and adopt healthy eating and physically activity behaviours [13-15]. The HEALTHY study in the U.S. is one example of an initiative where social-marketing based communications were integrated into a school program to reduce type-2 diabetes in children and where several indices of obesity showed reductions [16,17]. However, we are not aware of any other studies that have used a mix of television and other forms of mass media to support a comprehensive large scale multi-setting childhood obesity prevention program.

The Good for Kids program was one of several concurrent child obesity prevention strategies implemented in NSW following a 2002 Obesity Summit that set the state agenda for obesity prevention efforts. At the time of its initiation, the program represented Australia’s largest ever community based child obesity prevention program. Following a competitive NSW Government selection process, core funding of $1.5 million per annum (2006–2010) was provided to the Hunter New England Area Health Service (HNEAHS) to conduct a dissemination program that addressed child overweight and obesity and contributed to evidence for policy and practice in the HNE region of NSW, Australia. The multi-setting program was implemented in partnership with a broad range of government, non-government and private organisations. To increase effectiveness, the program linked setting-based policy and practice change with awareness raising. Given the complexity of the intervention design, it was not possible to attribute the behavioural changes observed in Good for Kids directly to the social marketing components. However, we were able to make a direct link between the communication campaign and awareness. The objective of this study was to describe the effectiveness of the Good for Kids communication campaign in raising awareness of child obesity prevention messages among parents and carers of children and to describe demographic associations with awareness.

## Methods

### Design

A controlled pre-post study was undertaken to evaluate awareness of the child obesity prevention program and its communication campaigns and messages using repeat cross-sections of an existing cohort of parents. Written informed consent was obtained from all parents and the program and its evaluation were approved by the Aboriginal Health and Medical Research Council (637/08) and the HNE Human Research Ethics Committee (HNEHREC 06/07/26/4.04).

### Setting

The study was set in the Hunter New England region of New South Wales, Australia. The region is geographically large with a diverse population of approximately 180,000 children aged up to 15 years. Its population resides in metropolitan, urban and suburban areas, regional centres, and rural and remote communities [18]. The region includes pockets of wealth and poverty, and has an overall socio-economic status that is lower than the state average [14]. The region incorporates areas of high population growth as well as areas with declining populations. Twenty-two per cent of New South Wales’ Aboriginal and Torres Strait Islander children live in the region [19].

### Sample

**Figure 1:**
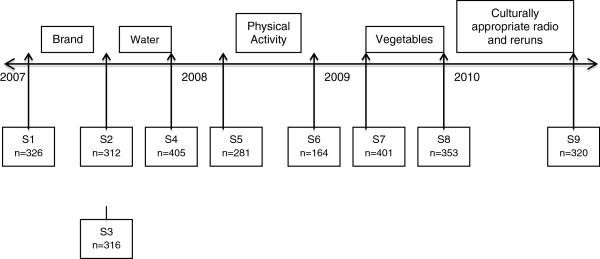
Timeline showing Good for Kids media campaigns and awareness surveys.

Parents of children aged 2–15 years across NSW who had participated in a baseline random digit telephone survey for the program and who agreed to be followed up at a later date were eligible to participate in the study (n = 1,594). Parents in this cohort were randomly selected for a series of repeat cross-sectional telephone surveys conducted before and after each of the communication campaigns. There were nine surveys in all corresponding to five mass media campaigns that were implemented during the program. The cohort was recycled from survey five onwards (Figure 1).

Approximately half of the participants in each survey were in the intervention group (those who resided within the HNE region) and the remainder were in the comparison group (those who resided in NSW but outside of the HNE region).

### Multi-setting child obesity prevention program

The Good for Kids program targeted children 2 to 12 years of age and was implemented in partnership with a broad range of government, non-government and private settings including child care services, schools, health services, sporting organisations and Aboriginal organisations. The program supported policy and practice changes conducive to healthy eating and physical activity among children. Five years after its commencement, and compared to the remainder of the state, the program was associated with: a significantly greater adoption of such policies and practices by settings in the HNE region and a significant reduction in girls 5–10 years who were overweight/obese.

### Good for kids social marketing strategy

The Good for Kids social marketing strategy was built around key behaviour change messages that were consumer tested and aired via TV, radio, print and other media such as childcare and school newsletters. The key messages and the timing of their delivery coincided with the long term behaviour change objectives of the program: drinking water instead of sweetened drinks; increasing physical activity; reducing sedentary behaviour; and increasing vegetable and fruit consumption. These messages were summarised in an initial branding campaign and promoted more extensively in separate campaigns for each message. The messages and their delivery were developed and planned using social marketing theory and focus group testing [20-22].

### Partnerships and brand development

In September 2006, a social marketing workshop was held to establish the fundamental features of the Good for Kids strategy including program name, brand, messages and imagery. During the workshop, key stakeholders reviewed issues relating to childhood obesity, healthy eating and physical activity as well as existing social marketing strategies focused on similar subjects. Stakeholders considered the goals and objectives of the Good for Kids program, discussed key messages which had the potential to counter possible barriers to change, identified target audience segments and identified the most suitable channels for the implementation of the strategy. Information from the workshop became the basis of a product description brief that was sent to three commercial creative agencies. Agencies were invited to submit proposals to create the name, brand, messages and imagery for Good for Kids. The proposals received were tested in target audience focus groups including Aboriginal communities. A panel of program staff and stakeholders selected the successful proposal based on how well it captured the intent of the program. In the focus group testing conducted by a market research agency, the proposal that presented the “Good for Kids” concept was found to the most convincing and motivating for both parents/ carers and children aged 12–15 years.

### Target audience

The target audience was segmented into children up to 12 years of age (~ 144,000); Aboriginal children up to 12 years of age and; parents and carers (grocery buyers aged 25 to 54 years) in the Hunter New England region of NSW. Grandparents, childcare workers, school teachers, sports coaches and health professionals and community members whose actions and advice influence children’s choices were a secondary target audience and were also considered in the development of the social marketing strategy.

### Message delivery

Good for Kids branding and messages were delivered to the target audience via five campaigns from 2007 to 2010 (Table 1). The campaigns included 30 and 15 second radio and television advertisements, channels chosen to maximise the exposure of the primary and secondary target groups. In the first campaign, print media (paid and unpaid) in prominent local and regional newspapers was also used to reach parents. All Good for Kids media was subject to government peer review and all campaigns, excluding one, were tested with Aboriginal and non-Aboriginal target audience focus groups (8–10 per group conducted in rural and urban parts of the region) prior to going to air.

**Table 1:** Good for kids media campaigns

**Campaign**	**Content**	**Key message(s)**
Brand	Children playing, drinking water and dressed up in vegetable costumes with a 3-step message jingle (1. Get active, get out and play, it’s good for kids one hour a day, 2. Drink H2O, think water first, 3. Two serves of fruit and five of vegies).	Drinking water instead of sweetened drinks, increasing physical activity and reducing sedentary behaviour and increasing vegetable and fruit consumption and reducing consumption, of energy dense nutrient poor foods. Launched the Good for Kids. Good for Life brand.
Water	Images highlighting the high amounts of sugars in soft drinks and fruit juices, and of children having fun drinking water.	Drinking water instead of sweetened drinks.
Physical activity	Children having fun being physically active, and getting bored watching TV/playing electronic games.	Children need 1 hour of physical activity everyday. Children should not play sitting down for more than 2 hours a day.
Vegetables	Images of parents serving up delicious and affordable vegetables in fun and creative ways.	It can take up to 10 times for kids to try vegetables before they like them.
Reruns	Previous campaigns were repeated.	All the above.

Good for Kids partnered exclusively with one media company to broadcast the television advertisements. Of the five free-to-air television stations that cover the region, the company selected provided the largest reach into the target audience and its broadcast footprint covered the entire Hunter New England region. The partnership arrangement included increased opportunities to reach the audience through Community Service Announcements (CSAs) which doubled the program’s television advertising placements;. The peak to off-peak split for Good for Kids TVCs was 70:30 and 60:40 for the rerun campaign. Similar deals and coverage arrangements were sought, and in most cases achieved, with radio and print media outlets across the region. Table 2 summarises the media buy and scheduling of each of the campaigns.

**Table 2:** Number and scheduling of good for kids advertisements for each campaign *

**2007**	**June**				**July**					**August**				**September**				
	3	10	17	24	1	8	15	22	29	5	12	19	26	2	9	16	23	30
***Good for kids brand***																		
TV (Sun-Sat)				200	150	150	100	100										
Radio (Sun-Sat)				15	15	15	15											
Print (Mon-Sat)				15	15	15	15	14										
***Water campaign***																		
TV (Sun-Sat)												200	150	100	100	100		
Radio (Sun-Sat)												15	15	15	15			
Print (Mon-Sat)												15	15	15	15	14		
**2008**	**June**				**July**					**August**				**September**				
	01	08	15	22	29	6	13	20	27	03	10	17	24	31	7	14	21	28
***Physical activity campaign***																		
TV (Sun-Sat)				80	70	70			80	70	70							
Radio (Sun-Sat)				60	60	60	60	60	60	60	60	60						
Print				Emails, posters, stakeholder newsletters														
**2009**	**September**				**October**					**November**				**December**				
	6	13	20	27	4	11	18	25		1	8	15	22	29	6	13	20	27
***Vegetables campaign***																		
TV (Sun-Sat)				180	200		100	100		100								
Radio (Sun-Sat)				136	136		136	136		116								
Print				Emails, posters, stakeholder newsletters														
**2010**	**February**				**March**					**April**				**May**				
	7	14	21	28	7	14	21	28	4	11	18	25	2	9	16	23	30	
***Culturally appropriate radio campaign***																		
Radio (Sun-Sat)		32	32	32	32	32	32	32	32									
***Re-run campaign***																		
TV (Sun-Sat)				105		105			105									
Radio (Sun-Sat)		80		80		80			80									

^*^Excludes community service announcements (CSAs) and bonus activity.

In addition to advertising activity, the program also benefited from branded promotional items such as water bottles; magnets; stickers; temporary tattoos; tennis balls; healthy shopping list templates; and various forms of physical activity equipment. These items were devised to encourage and support positive behaviour change. Good for Kids also gained regular editorial coverage throughout the regions many media outlets and had a presence at various community events in the Hunter New England region.

In 2009, towards the end of Good for Kids, a culturally appropriate communication campaign targeting Aboriginal audiences was developed. This campaign relied on direct communication activity, as well as a series of radio advertisements covering Good for Kids program messages. The radio campaign was aired through stations and programming for Aboriginal audiences. Due to the costs associated with finding a sufficiently large sample of people in this niche market using computer-assisted telephone interviews, the campaign was not formally evaluated.

### Other campaigns

Other communication campaigns promoting healthy eating and physical activity and sponsored by government were running during the intervention period in both the intervention and comparison areas and may have also played a role in raising awareness of physical activity and nutrition issues in the Hunter New England region. This included the ‘Get Moving’ campaign (2006) funded by the Federal Government, which sought to encourage children to be more active and NSW Health ran campaigns promoting fruit and vegetable consumption (2007) and water consumption (2008) [6,7,16].

### Data collection

Questions were developed by Good for Kids staff to evaluate the communication campaigns using computer-assisted telephone interviews. They included measures of socio-demographics, program awareness, campaign awareness, and awareness of campaign messages. Program awareness was assessed using a prompted recall question that asked participants if they had ‘…recently seen, read or heard anything about the Good for Kids program in the media?’ (yes/no). The question covered all forms of media implemented. Campaign awareness was assessed using questions that drew on the content of the advertisement to prompt recall. For example, the TV awareness question for the water campaign was, ‘Do you remember recently seeing a television advert with up beat music and lyrics with images of glasses being filled with juice and water?’ (yes/no). For campaign awareness, only TV is reported. To assess awareness of specific campaign messages, a range of potential messages were read out and participants were asked to select the correct one. Correct messages were that children should: drink water instead of sweetened drinks; be more active in play and sport and cut down on small screen recreation and; eat more vegetables and fruit.

### Statistical analysis

Analyses were performed using SAS software Version 9.2 (SAS Institute Inc., Cary, NC, USA). Logistic regression within a generalised estimating equation framework was used to assess change in program, campaign and message awareness over time in HNE compared to NSW. For each region, a model was run to assess change from pre- to immediately post-campaign and from pre-campaign to the end of the program. Models had terms for time, region and the interaction of time and region. The p-value from the interaction term was used to determine if there was a statistically significant change in awareness between regions over time. The regression models adjusted for differences in socio-demographic characteristics (country of birth, ethnicity (Aboriginal/Torres Strait Islander or non-Aboriginal), educational attainment and rural living) between the regions. Data from the final telephone survey was used to assess bivariate associations between program awareness and awareness of each of the campaigns with the socio-demographic characteristics of gender, age (<40 years cf. >40 years), locality, (urban cf. rural), education (tertiary cf. secondary or lower) and ethnicity (Aboriginal and Torres Strait Islander cf. non-Aboriginal) using chi square. All statistical tests were 2-tailed with an alpha of 0.05.

## Results

### Sample

Of the 1,595 parents or carers who agreed to be called back at the initial survey (98% of original consenters, n = 1,631), 1367 (n = 748 intervention; n = 619 control) participated in the surveys, giving an overall response rate of 86%. The majority of participants in the telephone surveys were female (80%-87%). For most surveys, HNE participants were significantly (p < 0.05) more likely to have been born in Australia, to be living in a rural area and to have lower levels of educational attainment than their NSW counterparts (Table 3). These findings were consistent with population data for the state [17]. HNE participants were also more likely to identify as Aboriginal or Torres Strait Islander and this difference was significant (p < 0.05) in survey four where there was a larger number of participants from HNE, reflecting the large proportion of Aboriginal and Torres Strait Islanders living in the region.

**Table 3:** Characteristics of telephone survey participants

**Characteristics**^**+**^	**Survey 1 **^**a**^		**Survey 2 **^**a**^		**Survey 3 **^**a**^		**Survey 4 **^**b**^	
	**HNE**	**NSW**	**HNE**	**NSW**	**HNE**	**NSW**	**HNE**	**NSW**
	**(n = 168)**	**(n = 158)**	**(n = 157)**	**(n = 158)**	**(n = 156)**	**(n = 165)**	**(n = 267)**	**(n = 138)**
	**%**	**%**	**%**	**%**	**%**	**%**	**%**	**%**
Female	84.5	84.2	79.6	81.0	83.3	82.4	84.6	87.7
Age								
<20 years	0.6	0.6	0	0	0.6	0	0	0
20-39 years	52.8	47.5	55.4	48.7	51.9	54.5	54.3	51.1
≥ 40 years	50.3	51.9	44.5	51.3	47.4	45.4	45.7	48.9
Country of birth								
Australia	91.7*	69.6*	90.4*	74.7*	93.6*	75.1*	87.3*	76.8*
Aboriginal/Torres Strait Islander status	3.6	1.3	3.2	3.2	3.2	2.4	5.1*	1.1*
Educational attainment								
Tertiary ^c^	58.3*	70.9*	56.0	61.4	52.6*	64.8*	62.6	68.1
Geographic location								
Rural ^d^	28.6*	12.8*	37.2*	12.7*	31.6*	17.2*	30.4*	13.2*
Number of children (mean (sd)) ^e^	1.9 (0.9)	1.8 (0.8)	1.9 (0.8)	1.9 (1.0)	1.8 (0.9)	1.9 (0.8)	1.9 (0.9)	1.9 (0.9)

^**+**^ Survey’s 5–9 not reported as cohort was recycled for these surveys.

^a^ 175 intervention and 175 comparison participants invited to participate at time 1–3.

^b^ 300 intervention and 149 comparison participants invited to participate at time 4.

^c^ Tertiary = TAFE certificate or diploma, University CAE or other tertiary institute qualification.

^d^ As defined by ARIA.

^e^ Number of children aged 2–15 years.

* Significant difference between intervention and control group p < 0.05.

### Program awareness

**Figure 2:**
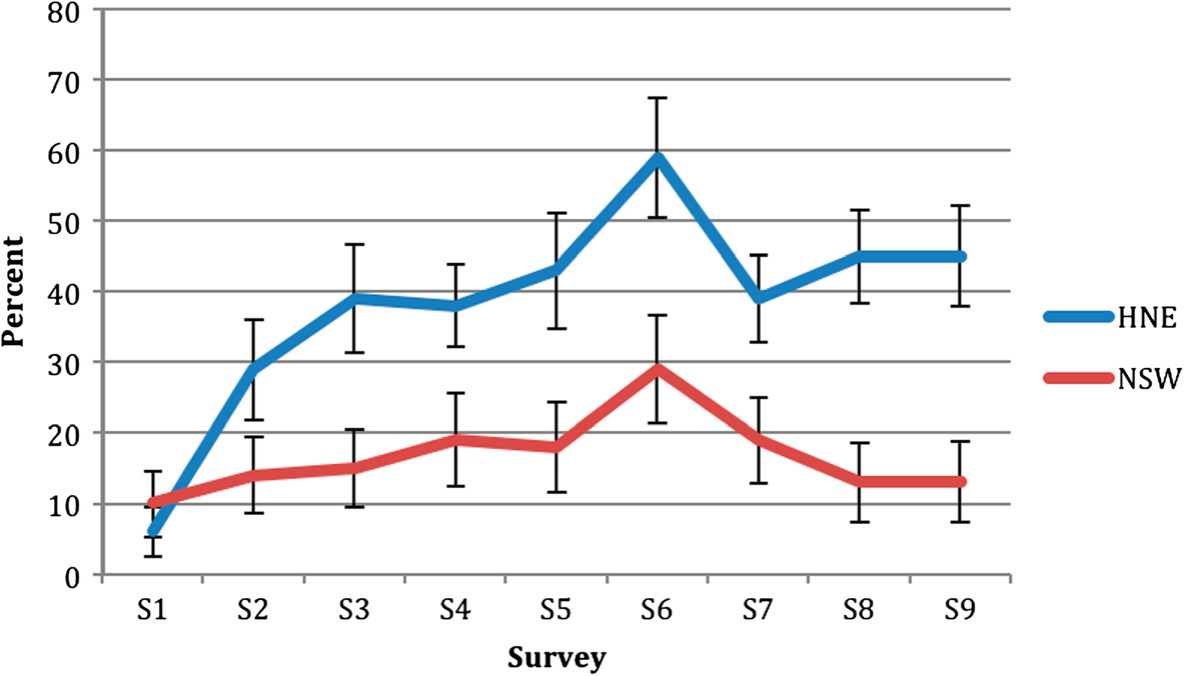
Awareness of Good for Kids in Hunter New England (HNE) compared to New South Wales (NSW) with unadjusted 95% confidence intervals.

After the first communication campaign (Figure 2), HNE participants (29%, 95% CI (22%-36%)) were significantly more likely to have seen, read or heard about the Good for Kids program in the media compared to those in the rest of NSW (14%, 95% CI (9%-19%) p = 0.01). Program awareness remained significantly higher (p < 0.001) in HNE at each subsequent survey with awareness peaking at 59% (95% CI, 50%-67%) in survey six. Change in program awareness between the regions over time was only significantly different at survey two and close to significance at survey eight (p for interaction term = 0.002 and 0.06 respectively). This indicates that after an initial increase in awareness in HNE, changes in awareness in HNE and NSW followed parallel pathways before diverging again between survey seven and eight. Change over time from baseline (survey one) to the end of the program (survey nine) in HNE (6% to 45%) was significantly greater than that observed for NSW (10% to 13%) (p for interaction term < 0.0001).

Following the final media campaign, there were no significant differences in program awareness across the socio-demographic groups (p = 0.08 – 0.63 not shown).

### Campaign awareness

**Figure 3:**
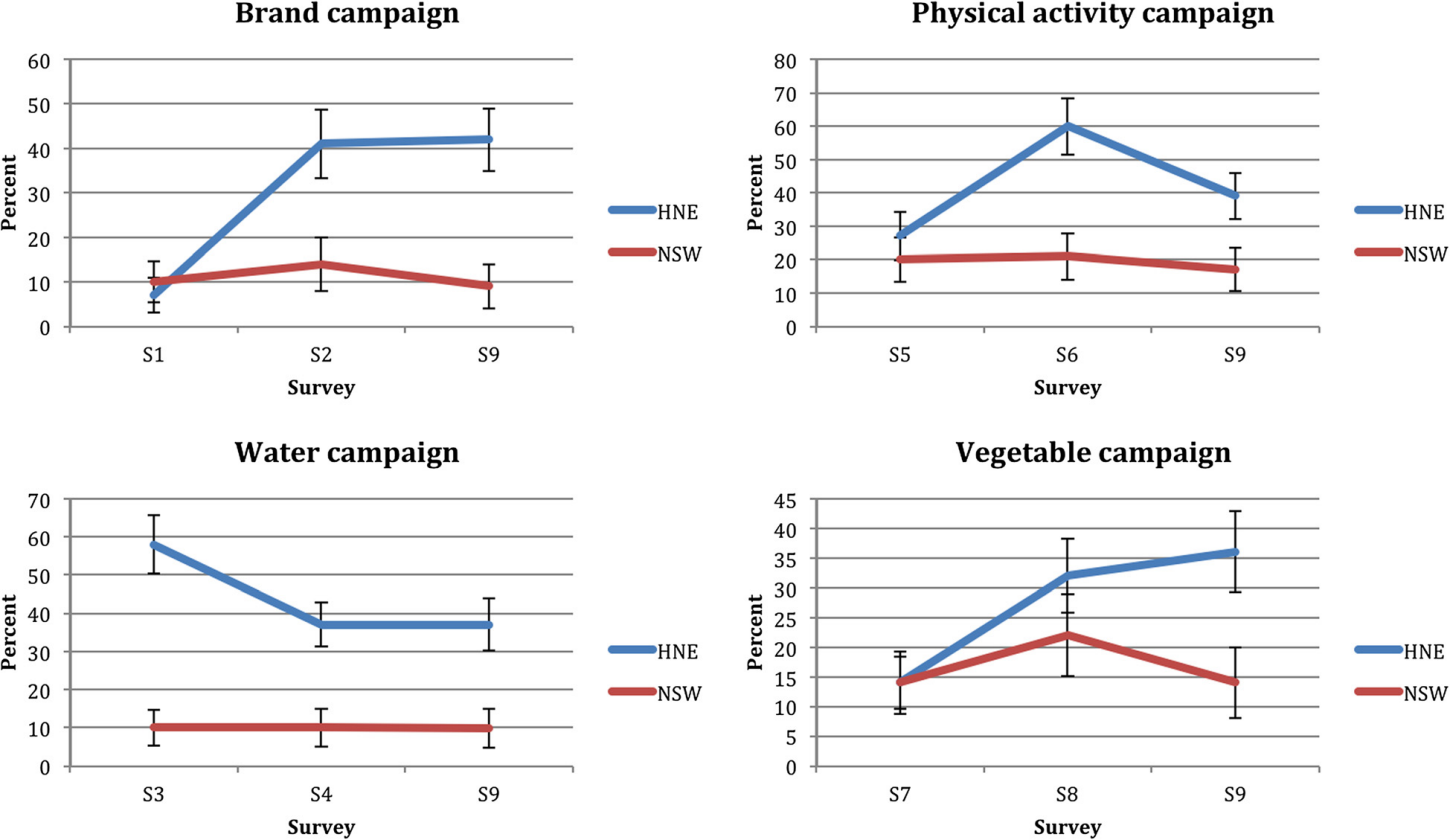
Awareness of the Good for Kids campaigns (unadjusted 95% confidence intervals).

The brand, water, physical activity and vegetable campaigns achieved 41%, 37%, 60% and 32% post-campaign awareness respectively in HNE compared to 14%, 10%, 21% and 22% in NSW (Figure 3). These differences were statistically significant (p < 0.0001) at each time point for all but the vegetable campaign (p = 0.10). By the end of the program however, awareness was significantly higher in HNE compared to NSW for all campaigns. In the final telephone survey, participants without a tertiary qualification were significantly more likely to have been aware of the Good for Kids brand (31% vs 20%, p = 0.04). There were no other significant differences in campaign awareness by socio-demographic group for the brand, (p = 0.17 – 0.68), water (p = 0.13 – 0.77), physical activity (p = 0.13 – 1.00) or vegetable (p = 0.31 – 0.80) campaigns.

With the exception of the water campaign, increases in awareness over time from baseline (survey one) to the end of the program (survey nine) were significantly greater in HNE compared to NSW (p for interaction terms < 0.05). In HNE, awareness of the water campaign decreased over time.

### Message awareness

**Figure 4:**
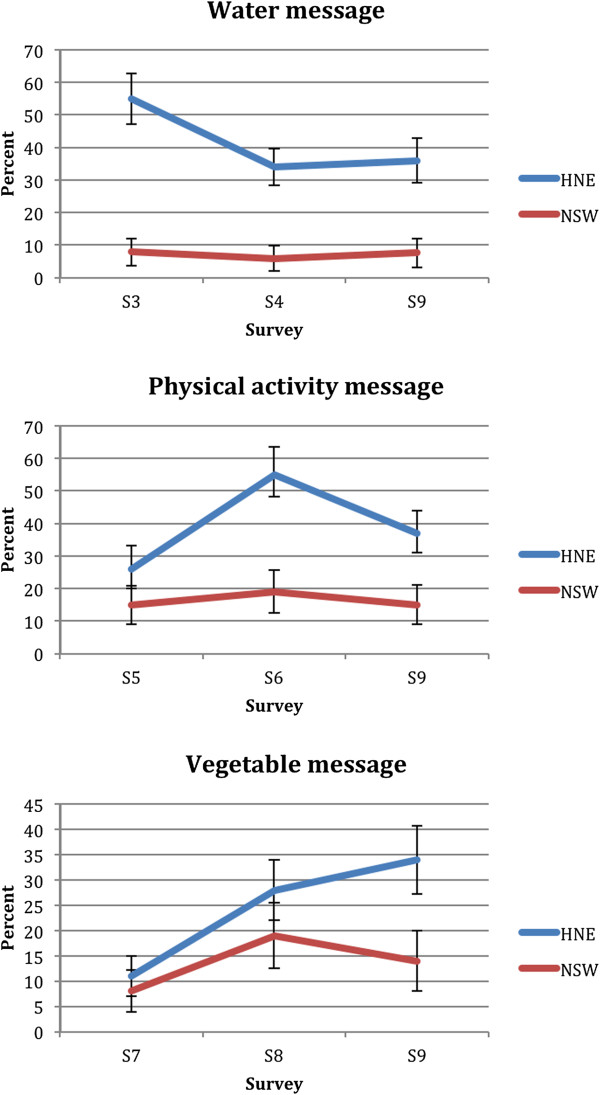
Proportion of participants who identified the main message of the water, physical activity and vegetable campaigns (unadjusted 95% confidence intervals).

The proportion of participants who correctly identified the main message of the water, physical activity and vegetable campaign is shown in Figure 4.

HNE participants were significantly (p < 0.001) more likely to identify the main water and physical activity messages, but not the vegetable message, immediately post-campaign compared to the rest of NSW participants. By the end of the program however, HNE participants were significantly more likely to identify all three key messages (p < 0.001). In spite of higher message identification in HNE, based on the time by region interaction terms, there were no significant differences in change over time in message awareness between the regions.

## Discussion

The findings show that the Good for Kids communication campaign effectively raised program awareness in the Hunter New England region. By the end of the program the brand, water, physical activity and vegetable campaigns were all effective and, after each campaign, parents in HNE were more likely than parents in NSW to identify the campaign messages that: children should drink water instead of sweetened drinks; be more active in play and sport, and cut down on small screen recreation and; eat more vegetables and fruit. There was also evidence that awareness of the program and campaigns reached all socio-demographic groups.

The awareness levels achieved in this study were similar to or higher than those achieved by other Australian government campaigns. For example, unprompted recall of the Get Moving campaign increased from 10% to 43%, [7] and recall of the NSW Go for 2 and 5 campaign increased from 46% to 68% [8]. Prompted recall of the NSW Health water campaign in 2008 increased from 19% to 53% [16]. It should be noted that the evaluation of this water campaign included parents from HNE where the same campaign had run earlier in the year and this may account for the higher NSW level of awareness at baseline (19%) compared to the level of awareness at baseline that was observed in the Hunter New England region (10%, see Figure 3). Evaluation of this campaign also found significant increases in parent’s awareness of the key messages, knowledge of the high sugar content of sweetened drinks (8% to 16%) and a decline in the reported consumption of sweetened drinks by boys and girls [16]. Unlike Good for Kids, however, these campaigns were not sustained and were not supported by accompanying strategies, such as healthy eating and physical activity policy and programs in preschools, schools, sports clubs and community service agencies to facilitate to the adoption of the changes being promoted. Also, with regard to their evaluation, because they were run state-wide they did not have comparison data to determine background awareness and it is thus not possible to determine the effectiveness of these campaigns. Finally, we were able to show that all socio-demographic groups had increased awareness, demonstrating equity in the delivery of the program and campaigns.

Other programs targeting obesity have also used mass media to raise awareness, achieving similar or higher levels of awareness than observed in Good for Kids. The United States Centres for Disease Control used a multi-setting social marketing campaign called VERB to promote physical activity among youths (aged 9 to 13 years) nationally [18]. Campaign awareness was 81% after two years of implementation. In 1998, the New South Wales government conducted a state-wide mass-media campaign to promote regular moderate-intensity physical activity among adults aged 25 to 60 who were motivated to increase their levels of physical activity but were insufficiently active [23]. This campaign successfully increased unprompted and prompted recall of physical activity messages to 21% and 51% of the target population respectively.

To achieve high levels of community awareness, Hornik and Kelly emphasize the importance of obtaining high levels of exposure for messages [24]. They note that paying for exposure is the only way of guaranteeing sufficient air time but that exposure can also be donated by media companies, for example through CSAs, and earned through editorial media coverage, or ‘making news’. Good for Kids earned editorial exposure through regular communication and building strong relationships with local media outlets, creating news through making program announcements, releasing research findings, holding community and media events and offering strong photo opportunities. Good for Kids program messages were also delivered via print media such as through newsletters and special publications distributed throughout the settings where program interventions were conducted (school newsletters, childcare centre websites etc.). The program also linked with parent’s networks and other relevant organisations to deliver program messages. For example, the Good for Kids vegetable campaign won the 2010 national Parents Jury (http://www.parentsjury.org.au) award for the best marketing campaign to promote healthy eating or physical activity to children. In addition, all program communication included an action point for the audience to visit the program website for further resources and information. The website http://www.goodforkids.nsw.gov.au played a crucial role as a platform for the many program audiences and stakeholders to interact with the program. The program website was also linked to and from other relevant stakeholder sites to gain further exposure for the program.

By using a mix of media (TV, radio, print, web and others) as well as donated (CSAs) and earned exposure (news releases and other contributions to local media outlets), Good for Kids partly overcame the limitation of having its television advertisements aired on only one station. Based on an independent assessment of the campaigns by Mediacom (a Sydney based media company) the target audience rating points (TARPS) for each of the campaigns achieved or exceeded (by 45-50%) what was planned for based on that anticipated by the media expenditure (data not shown). This meant that, due to CSAs, more of the target group of grocery buyers aged 25–54 years were exposed to the television advertisements than was paid for by the program. In accord with communication [25] and social marketing principles, [12] the Good for Kids program aimed to have a clearly defined target audience. Due to budget constraints that limited capacity to advertise the messages across a large number of segments, the program developed advertising that would appeal to target audience that included children and their parents. However, segmentation was facilitated within this audience using child and parent specific support materials (such as water bottles for kids and newsletters for parents) and some campaigns were directed more at kids (eg water campaign) or parents (eg vegetable campaign) depending on who was in the best position to act on the message. Formative research led to good message awareness by parents given the complexities associated with conveying simple and actionable diet and physical activity messages in an environment where people are exposed to competing messages from many sources [26,27]. Also, it was useful to get target audience groups to reflect on previous campaigns to help improve subsequent campaigns. A challenge for Good for Kids, was timing the advertising campaigns so they coincided with the policy and program changes in preschool, schools and other settings. Ideally, for example, messages promoting fruit and vegetable consumption would have coincided with the launch of ‘Crunch and Sip’ , an initiative which provided time for children to eat fruit and vegetables in schools. However, the logistics of implementing multiple strategies in a variety of settings prevented such synergies occurring. Also, in line with communication principles, Good for Kids used a variety of media to deliver key messages.

There are several limitations that should be kept in mind when considering these findings. Firstly, the campaigns were not controlled over time (i.e. one campaign may have influenced awareness of another). This may explain the high baseline awareness of the water campaign in Hunter New England region (Figures 3 and 4). The water campaign began two weeks after the brand campaign, which included a prominent message about drinking water. Because these campaigns were close together, participants may have recalled the earlier campaign and message and hence inflated campaign awareness. Secondly, from survey five onwards, there may have been panel conditioning where participants responses were influenced by the number of times they had taken the survey. After three surveys over three years, panel conditioning was found to inflate the percentage of respondents in a cohort who reported awareness of the VERB campaign by about 8.5% [28]. In our study, the overall sample and survey size meant each respondent was only likely to have been surveyed twice. Also, in all cases, differences in post-campaign awareness were greater than 10%. Thirdly, Good for Kids media and materials may have been seen in the comparison region, perhaps explaining the increase in program awareness among NSW participants (Figure 2). It is possible that news releases about the program contributed to this as ministerial releases about Good for Kids were regular and state-wide during the early years of the program. The 2008 NSW water campaign may also have increased comparison group awareness at survey 6 as this survey was conducted just as the campaign ended and the Good for Kids logo and tagline feature prominently in the water campaign advertisement. Additionally, the intervention and comparison areas shared a geographic boundary and there may have been some cross border contamination. Finally, the surveys had small sample sizes making small changes in program and campaign awareness difficult to detect. This also limited our ability to assess awareness among Aboriginal communities.

## Conclusions

Through the Good for Kids communication campaign, high levels of awareness were achieved and maintained and parents of all socio-demographic groups picked up the main messages. For future population-based childhood obesity prevention programs these finding suggest that media can be harnessed to increase awareness of healthy eating and physical activity messages, that media may assist in raising awareness among hard-to-reach groups, that a series of related campaigns can build program awareness and that despite their limitations, panel methods are a viable and cost-effective tool for campaign evaluation.

## Competing interests

The authors declare that they have no competing interests.

## Authors’ contributions

ACB contributed to the design of the study, study implementation and coordination and drafted the manuscript. LW, RS, LC, KY, RH and NO contributed to the implementation and coordination of the study and helped to draft the manuscript. MF performed the statistical analysis. AJM contributed to the development of the survey instruments and helped draft the manuscript. JW contributed to the design of the study, its implementation and coordination and helped to draft the manuscript. All authors read and approved the final manuscript.
